# SNORD63 and SNORD96A as the non-invasive diagnostic biomarkers for clear cell renal cell carcinoma

**DOI:** 10.1186/s12935-020-01744-4

**Published:** 2021-01-18

**Authors:** Xiaoling Shang, Xingguo Song, Kangyu Wang, Miao Yu, Shanshan Ding, Xiaohan Dong, Li Xie, Xianrang Song

**Affiliations:** 1 Department of Clinical Laboratory, Cheeloo College of Medicine, Shandong Cancer Hospital and Institute, Shandong University, Jinan, 250012 Shandong China; 2 Department of Clinical Laboratory, Shandong First Medical University and Shandong Academy of Medical Sciences, Shandong Cancer Hospital and Institute, 440 Ji-Yan Road, Jinan, 250117 Shandong People’s Republic of China; 3 Department of Clinical Laboratory, Shandong Provincial Third Hospital, Cheeloo College of Medicine, Shandong University, Jinan, 250031 China

**Keywords:** SNORD63, SNORD96A, Urinary sediment, ccRCC, Diagnostic biomarkers

## Abstract

**Background:**

Increasing evidence has demonstrated that snoRNAs play crucial roles in tumorigenesis of various cancer types. However, researches on snoRNAs in ccRCC were very little. This study mainly aimed to validate the differential expression and the potential diagnostic value of SNORD63 and SNORD96A in ccRCC.

**Methods:**

SnoRNAs expression was downloaded from the SNORic and TCGA database including 516 patients with ccRCC and 71 control cases. SNORD63 and SNORD96A expression were further detected in 54 tumor and adjacent FFPE ccRCC tissues, 55 plasma and 75 urinary sediment of ccRCC patients. Then, differential expression and diagnostic value of SNORD63 and SNORD96A were further calculated.

**Results:**

SNORD63 and SNORD96A expression were significantly increased in ccRCC tissues compared with normal tissues from the TCGA database (both, *P* < 0.0001). In addition, we found that SNORD63 and SNORD96A localized in plasma and US stably after treating with RNase A. Meanwhile, SNORD63 and SNORD96A in FFPE and US were elevated in ccRCC patients (all, *P* < 0.0001). However, plasma SNORD63 expression had no significance while SNORD96A significantly increased in plasma of ccRCC patients. Notably, the AUC of SNORD63 in US was 0.7055, by comparison the AUC of plasma SNORD63 was only 0.5161. However, the AUC of plasma SNORD96A was up to 0.8909, by comparison the AUC of SNORD96A in US was 0.6788. Interestingly, the AUC of plasma SNORD96A in early stage ccRCC was highly up to 0.9359.

**Conclusions:**

Our findings revealed that SNORD63 in US and SNORD96A in plasma could act as the promising non-invasive diagnostic biomarkers for ccRCC patients.

## Background

Clear cell renal cell carcinoma (ccRCC), the most prevalent histologic subtype accounting for 90% of renal cell carcinoma (RCC), is one of the most common urological malignancy in adults [1, 2]. Due to the lack of dramatic symptoms and specific biomarkers, 30% of ccRCC patients develop metastasis at the time of initial diagnosis, increasing the difficulty of treatment and hindering further improvements in the cure rate [3]. Therefore, it is urgent to identify sensitive and reliable biomarkers for ccRCC diagnostics.

Small nucleolar RNAs (snoRNAs), are a family of conserved nuclear RNAs (60–300 nt), widely exist in Cajal bodies or nucleolus of eukaryotic cells [4], where they guide post-transcriptional modification of rRNAs and some spliceosomal RNA [5, 6]. In recent years, accumulating evidence suggest that snoRNAs play a crucial role in the tumorigenesis and tumor development [7], aberration of their expression has been observed in multiple many cancers, some of which are cancer type-specific. A pan-cancer analysis of snoRNAs expression landscape identified 46 individual snoRNAs that have clinical relevance and which may play significant roles in tumorigenesis [8]. Besides, a research has established a risk assessment model based on a six-snoRNA signature, they proved ccRCC patients in high-risk group had significantly shorter overall survival and recurrence-free survival than those in low-risk group. Interestingly, this six-snoRNA signature had the capability to act as an independent and superior diagnosis prognosis indicator for ccRCC [9].

SnoRNAs are stably expressed and measurable in body fluids including the blood plasma, serum, and urine of cancer patients [10], empowering snoRNAs with the potential as the non-invasive biomarkers for diagnostics of malignancies. It has been reported that SNORD33, SNORD66, SNORD76 could be the potential biomarkers for non-small cell lung cancer (NSCLC). The kidney is the organ that produces urine in human body. During normal urination, a small amount of kidney cells is exfoliated into the urinary sediment (US), whereas urinary malignance could facilitate this exfoliation. US sampling is a favorable non-invasive choice for detection of snoRNAs for ccRCC diagnosis. This is because US contains cancerous or precancerous kidney cells. These data suggest the snoRNAs in circulation and US may act as the non-invasive biomarker for ccRCC diagnostics.

In our study, we firstly screened out SNORD63 and SNORD96A, then validated their differential expression based on the study from The Cancer Genome Atlas (TCGA), SNORic database and formalin fixed paraffin embedding (FFPE) samples, plasma and US, implying the potential role in tumorigenesis. Importantly, SNORD63 in US and SNORD96A in plasma possessed the favorable diagnostic efficiency, suggesting that aberrant expression of SNORD63 and SNORD96A act as diagnostic promising biomarkers for ccRCC.

## Materials and methods

### Data source

The snoRNA gene expression data of ccRCC tumor and adjacent tissue samples was downloaded from the SNORic database (http: //bioinfo.life.hust.edu.cn/SNORic) [8] and its corresponding clinical information were obtained from TCGA (http://cancergenome.nih.gov). A total of 516 ccRCC patients and 71 control cases between 2011 and 2015 were included in this study.

### Patients, healthy donors and samples collection

Urine samples were obtained from total 75 ccRCC patients admitted to Shandong Cancer Hospital and Institute from April 2019 to December 2019, as well as from total 96 healthy donors admitted to Shandong Provincial Third Hospital at May 2019; FFPE samples of ccRCC tissue and paired para-cancerous tissue were collected from total 54 ccRCC patients admitted to Qilu Hospital of Shandong University between 2018 and 2019.

Plasma samples were obtained from 55 ccRCC patients and 40 healthy donors admitted to Shandong Cancer Hospital and Institute from April 2019 to June 2020. ccRCC patients were diagnosed by the combination of clinical, pathological, radiological approaches, the tumor stage was determined according to the American Joint Committee on Cancer (AJCC) eighth edition TNM stage and grade were determined according to the 2016 edition World Health Organization (WHO) Furman grading system; All patients didn’t receive any anti-tumor treatment before sampling, or suffer any other endocrine, immune, or metabolic diseases. The healthy donors did not present any disease. Informed consent was obtained for all individuals.

Isolation of US was performed as described previously [11]. Total 15 ml fresh morning urine was collected and centrifuged at 3000*g* for 20 min at room temperature followed by PBS washing three times. The sediment was collected and stored at − 80 °C; Peripheral blood samples were collected into EDTA tubes and centrifuged at 3000*g* for 10 min at 4 °C to separate from the peripheral blood cells, followed by another 12,000*g* centrifugation for 10 min at 4 °C to pellet any remaining cells. The supernatant was collected and stored at − 80 °C.

### Cell culture

Human renal cancer cell lines ACHN, 786-O, Caki-1 and OS-RC-2 were purchased from China Center for Type Culture Collection (Wuhan, China) and cultured in RPMI 1640 (Gibco, Invitrogen, Carlsbad, CA, United States). All cell lines were supplied with 10% fetal bovine serum (FBS, Gibco), 1% penicillin and streptomycin and incubated at 37 ℃ in a 5% CO_2_ humid incubator.

### RNA isolation

Total RNAs of FFPE samples were extracted using miRNAprep Pure FFPE kit (Tiangen Biotech, Beijing, China) according to the instructions of the kit; Briefly, repeated dewaxing with xylene and anhydrous ethanol was performed in a collection tube, then proteinase K and buffer RF were added into the pellet followed by vortex to mix thoroughly and incubation. After equilibrium, Spin Column CR3 was put back to the collection tube, followed by incubation with Buffer RW for 2 min at room temperature, and finally elated by RNase free water. Total RNAs of each 250ul plasma was isolated by adding 750ul TRIzol® LS Reagent (Thermo Fisher Scientific, Carlsbad, CA, US) and total RNAs of US, cells and exosomes were extracted using 500ul TRIzol Reagent (Thermo Fisher Scientific, Carlsbad, CA, US) according to the manufacturer's protocol.

### Quantitative PCR (qPCR)

The 3 μg isolated RNA was reverse-transcribed into complementary DNA (cDNA) using Mir-X miRNA First-Strand Synthesis Kit (TaKaRa Bio, Nojihigashi, Kusatsu, Japan) in 10 μl reaction according to the manufacturer's instructions. Furthermore, qPCR was conducted by TB Green Premix Ex Taq II Reagent (TakaRa Bio, Nojihigashi, Kusatsu, Japan) in a volume of 20 μl (10 μl of TB Green Premix Ex Taq II Reagent, 0.4 μl of forward primer, 0.4 μl of reverse primer, 2 μl cDNA and 7.2 μl water) at the LC480 qPCR system (Roche Diagnostics, BALE, Germany). The reactions started at 95 °C for 30 s, followed by 45 cycles of 95 °C for 5 s and 60 °C 30 s; then ended at 50 °C 5 s and 60 °C for 1 min. All experiments were carried out in duplicate, and then the median Ct was calculated, U6 was used as an internal control. The relative expression of snoRNAs was evaluated by the comparative cycle threshold (ΔCt) method: (ΔCt = Ct^snoRNA^–Ct^U6^) as described previously [12]. The qPCR primers were listed in Table 1 and Additional file 1: Table S1.

**Table 1:** Primers sequence involved

Gene	Forward primer	Reverse primer
*SNORD63*	GTGCAATGATGTATTTTATTCAACACA	GCTCAGTCATTAGTTTTCCACACG
*SNORD96A*	CCTGGTGATGACAGATGGCA	CCTTCAGAATTGCAGGACATGT
*U6*	TGGAACGCTTCACGAATTTGCG	GGAACGATACAGAGAAGATTAGC

### Statistical analysis

Statistical analyses were carried out using Statistical Product and Service Solutions 22.0 software package (SPSS, Chicago, IL, USA) or GraphPad Prism version 8.0 (GraphPad Software, San Diego, CA, USA). The Kolmogorov–Smirnov test was carried out to check the normality of the distribution. If the data followed normal analysis, t test would be used; if not, Mann–Whitney test would be used. In paired data, the normally distributed numeric variables were evaluated by paired t-test, whereas non-normally distributed variables were analyzed by Wilcoxon rank-test. The receiver operator characteristic (ROC) analysis was used to determine the sensitivity, specificity and area under curve (AUC) was calculated to evaluate diagnostic efficiency. Data were shown as median ± interquartile range and *P* value < 0.05 was defined as statistically significance, and all tests were set as double-tailed.

## Results

### Identification of the differential snoRNAs in ccRCC from database

**Fig. 1:**
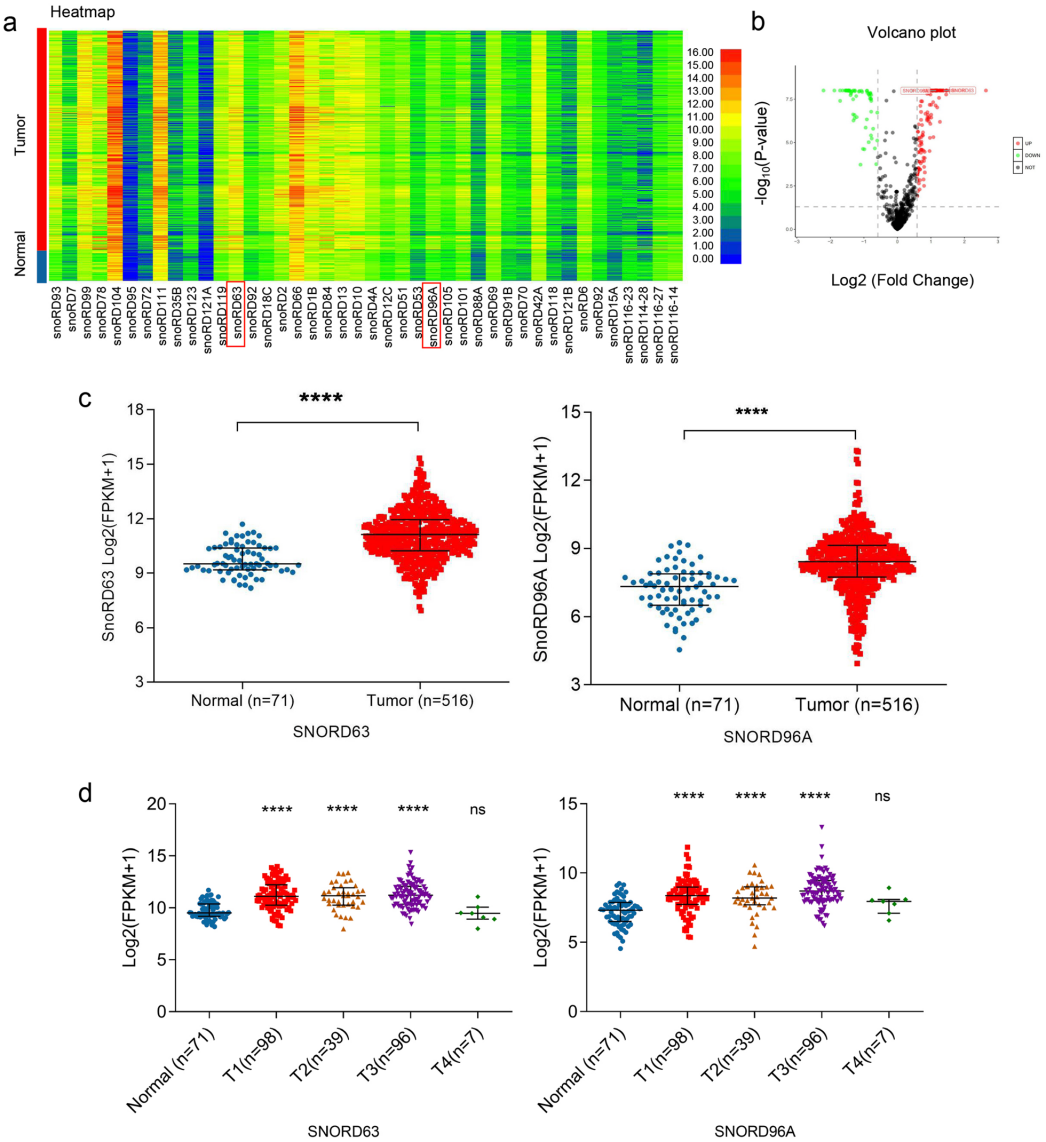
Identification of SNORD63 and SNORD96A differential expression in ccRCC. **a** The differentially expressed snoRNAs in SNORic database ccRCC tumor tissues (N = 516) and adjacent tissues (N = 71). **b** Volcano plot compared the expression fold-change of snoRNAs in ccRCC tissues vs. adjacent normal tissues. **c** The differentially expression of SNORD63 and SNORD96A in ccRCC tumor tissues and adjacent tissues using SNORic database (****P < 0.0001). D. The relationship between SNORD63, SNORD96A expression and different T stage (****P < 0.0001, ns: no significance)

To screen out the differential expression levels in ccRCC, snoRNAs in 516 tumor tissues and 71 control tissues and their clinical characteristics from the TCGA and SNORic database were analyzed. The upregulated and downregulated snoRNAs were screened in ccRCC tissues compared to those in control tissues. The expression patterns of these snoRNAs were shown as the heatmap using hierarchical cluster analysis (Fig. 1a) and volcano map (Fig. 1b). Due to the low primers’ specificity and expression level, some snoRNAs were ruled out and others were subjected to validation in FFPE tissues including 24 ccRCC and paired para-cancerous tissues. After that, SNORD104, SNORD111, SNORD95, SNORD63, SNORD96A and SNORD10 were selected as the candidates, further validated in US in a cohort with 24 ccRCC patients and 24 healthy donors. Unexpectedly, the expression of SNORD104, SNORD111, SNORD95 and SNORD10 were up-regulated in FFPE tissues (Additional file 2: Fig. S1A) but exerted no significant differences (Additional file 2: Fig. S1B) in US of ccRCC compared with those in healthy donors. Therefore, SNORD63 and SNORD96A were finally identified because of their significantly differential expression in FFPE and US.

Then, we analyzed the expression of SNORD63 and SNORD96A between ccRCC tissues and normal control. As shown in Fig. 1c, both SNORD63 and SNORD96A levels were significantly increased in tumor tissues compared to normal tissues (*P* < 0.0001). In addition, we also analyzed the correlation between the levels of SNORD63, SNORD96A and clinicopathological characteristics. As shown in Table 2 and Fig. 1d, when compared with the normal contol, the expression levels of SNORD63, SNORD96A were significantly up-regulated in T1, T2 and T3 stage but not in T4 stage, which largely attributed to the small T4 sample size (only 7).

**Table 2:** Relationship between clinical characteristics and SNORD63, SNORD96A expression in TCGA database

Variables	SnoRNAs	
	SNORD63	SNORD96A
Age		
< 61	0.5335	0.8183
≥ 61		
Sex		
Female	0.8384	0.7945
Male		
T stage		
T1	0.0087**	0.0129^*^
T2		
T3		
T4		
N stage		
N0	0.1997	0.3218
N1		
M stage		
M0	0.3788	0.6025
M1		
Grade		
G1	0.5213	0.4832
G2		
G3		
G4		

To further validate the expression of SNORD63 and SNORD96A, total RNAs of renal cancer cell lines were extracted. The result showed that SNORD63 and SNORD96A had relative expression in ACHN, 786-O, OS-RC-2 and Caki-1, higher in ACHN and 786-O but lower in Caki-1 and OS-RC-2. The specific expression of SNORD63 and SNORD96A in renal cancer cells were shown in Additional file 3: Figure S2.

### SNORD63 and SNORD96A localized in plasma and US stably

**Fig. 2:**
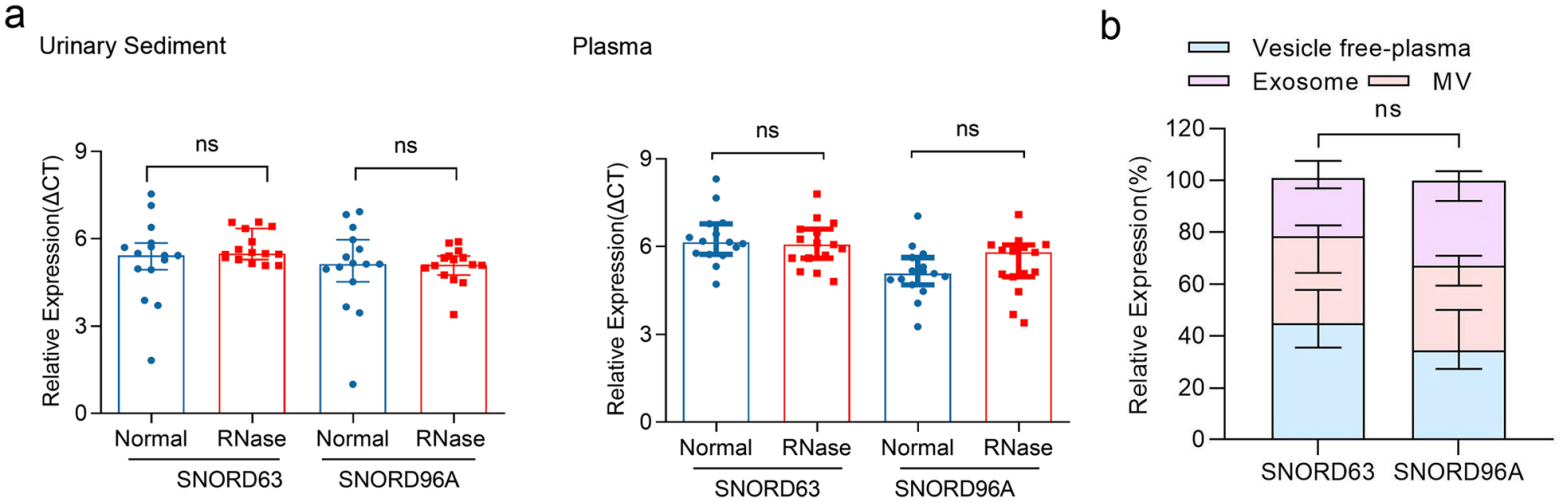
SNORD63 and SNORD96A localized in plasma and urinary sediment (US) stably. **a** The expression of SNORD63 and SNORD96A in US and plasma after RNase treatment (ns: no significance). **b** The location of SNORD63 and SNORD96A in plasma. (MV: microvesicles, ns: no significance)

The stability of SNORD63 and SNORD96A in plasma and US was detected. Firstly, US from 15 ccRCC patients and subjected to RNase treatment. As shown in Fig. 2a, no different expression of SNORD63 and SNORD96A in US was observed under RNase treatment. Similarly, RNase also failed to degrade SNORD63 and SNORD96A in plasma from 15 ccRCC patients (Fig. 2b). Next, we verified their position in plasma. As shown in Fig. 2c, both SNORD63 and SNORD96A exited in microvesicles (MV), exosome and vesicle free plasma, indicating their stability in plasma might not attribute to the protection by bilayer membrane. Unexpectedly, no difference of their position in plasma was observed.

### The expressoion of SNORD63 and SNORD96A in FFPE, plasma and US

**Fig. 3:**
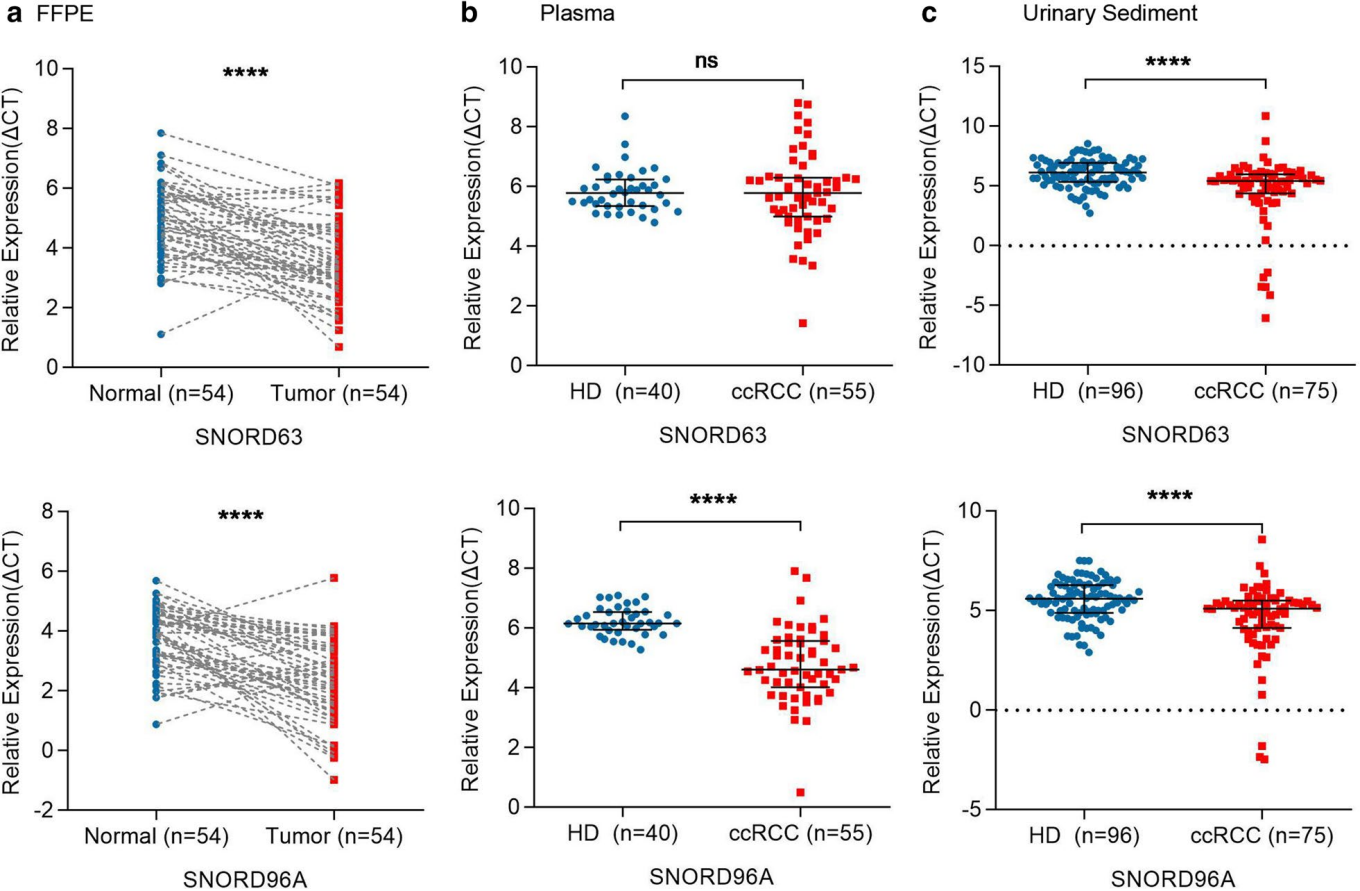
The expression of SNORD63 and SNORD96A in formalin fixed paraffin embedding (FFPE), plasma and urinary sediment (US) for ccRCC patients. **a** The differentially expression of SNORD63 and SNORD96A in FFPE in 54 ccRCC patients compared with adjacent tissues (****P < 0.0001). **b** The differentially expression of SNORD63 and SNORD96A in plasma in 55 ccRCC patients vs. 40 healthy donors (HD) (ns: no significance, ****P < 0.0001). **c** The differentially expression of SNORD63 and SNORD96A in US in 75 ccRCC patients vs. 96 healthy donors (HD) (****P < 0.0001)

We further evaluated SNORD63 and SNORD96A expression in 54 ccRCC FFPE and paired para-cancerous tissues. As shown in Fig. 3a, the expression of SNORD63 and SNORD96A were significantly increased in ccRCC tissues compared to normal tissues (*P* < 0.0001, both). Consistently, SNORD63 and SNORD96A in US were also significantly increased in ccRCC patients compared with those of healthy donors (*P* < 0.0001, both, Fig. 3b). Unexpectedly, SNORD96A expression was higher in plasma of ccRCC patients than in that of healthy donors (*P* < 0.0001), whereas SNORD63 in plasma seemed no significant difference between the two group (Fig. 3c).

We also analyzed the correlation between SNORD63, SNORD96A expression and clinicopathological characteristics. However, the result showed that they were not related to patients’ age, gender, T stage, N stage, M stage and grade (all, *P* > 0.05) other than the expression of SNORD63 in US was related to age (*P* = 0.0105) (Table 3).

**Table 3:** Relationship between clinical characteristics and SNORD63, SNORD96A expression in FFPE, US and plasma

Variables	FFPE		US		Plasma	
	SNORD63	SNORD96A	SNORD63	SNORD96A	SNORD63	SNORD96A
Age						
< 61	0.8527	0.5048	0.0105*	0.0654	0.1710	0.6177
≥ 61						
Sex						
F	0.7128	0.4462	0.9889	0.8299	0.7475	0.8117
M						
T stage						
T1	0.7994	0.8722	0.8722	0.060	0.7098	0.2791
T2						
T3						
T4						
N stage						
N0	0.6919	0.6257	0.7181	0.6384	0.2110	0.3544
N1						
M stage						
M0	0.6164	0.4695	0.8306	0.8709	0.9748	0.0930
M1						
Grade						
G1	0.3182	0.1568	0.1722	0.9956	0.4288	0.7813
G2						
G3						
G4						

### SNORD63 and SNORD96A as the non-invasive diagnostic biomarkers for ccRCC

**Fig. 4:**
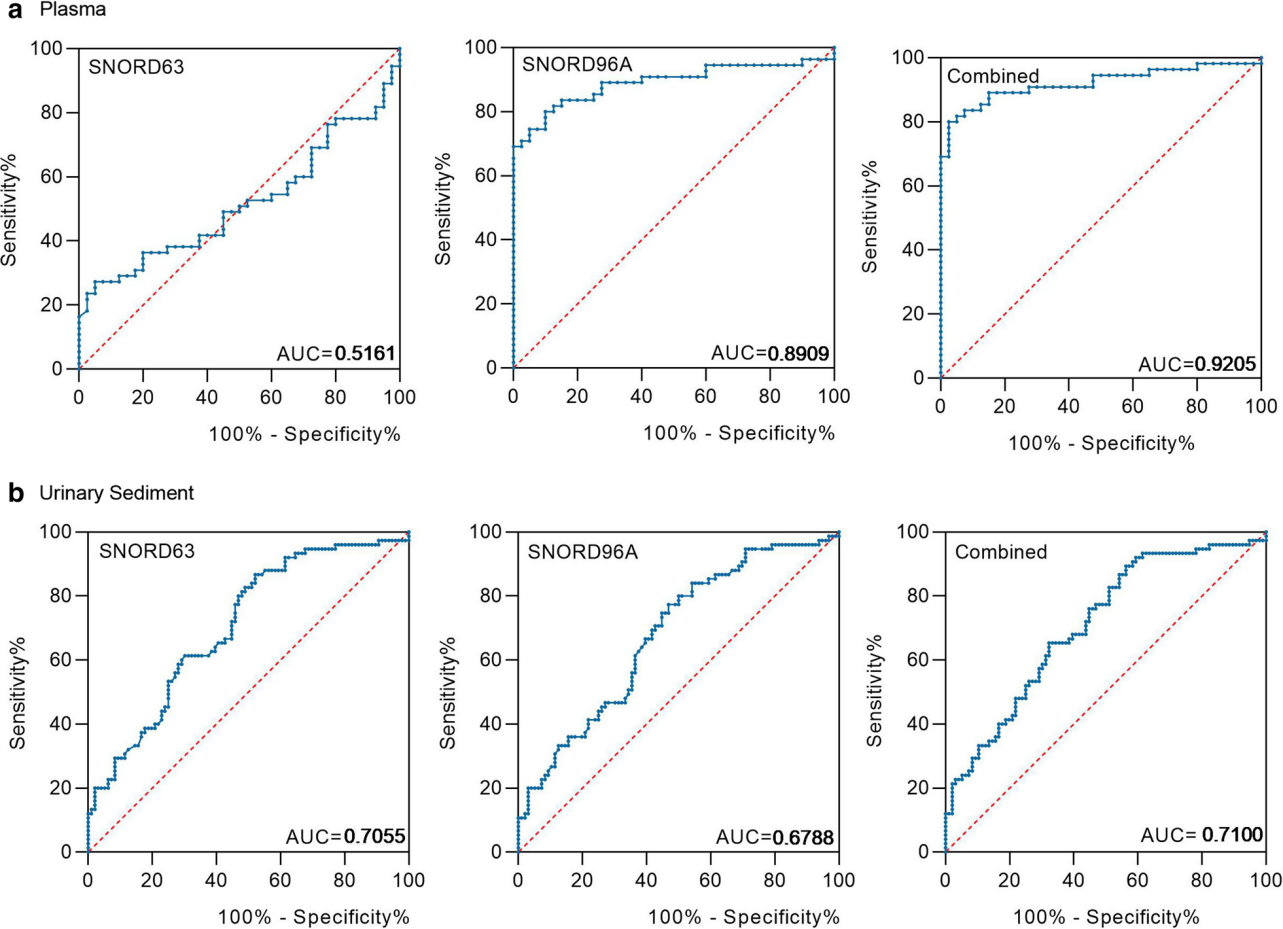
SNORD63 and SNORD96A as the non-invasive diagnostic biomarkers for ccRCC patients. **a** The areas under the curves (AUCs) of SNORD63 and SNORD96A in plasma were 0.5161 and 0.8909, respectively. The diagnostic performance for their combination demonstrated the AUC of 0.9205. **b** The areas under the curves (AUCs) of SNORD63 and SNORD96A in urinary sediment (US) were 0.7055 and 0.6788, respectively. The diagnostic performance for their combination demonstrated the AUC of 0.7100

To evaluate diagnostic performance of SNORD63 and SNORD96A for ccRCC, a receiver-operating characteristic (ROC) curve was calculated in US and plasma, respectively. As shown in Fig. 4a, in plasma, the areas under the curves (AUCs) of SNORD63 and SNORD96A were 0.5161 with 95% sensitivity and 27.3% specificity, 0.8909 with 90% sensitivity and 80% specificity, respectively. Besides, the diagnostic performance for their combination demonstrated the AUC of 0.9205 with a relative sensitivity of 80% and a relative specificity of 97.5%; In US, the AUCs of SNORD63 and SNORD96A were 0.7055 with 47.9% sensitivity and 86.7% specificity, 0.6788 with 53.1% sensitivity and 77.3% specificity, respectively. Moreover, the diagnostic efficiency of their combination was also calculated, processing AUC of 0.7100 with a relative sensitivity of 43.7% and a relative specificity of 89.3% (Fig. 4b), indicating SNORD63 and SNORD96A act as the promising non-invasive diagnostic biomarkers for ccRCC.

### SNORD63 and SNORD96A as the early diagnostic biomarkers for ccRCC

**Fig. 5:**
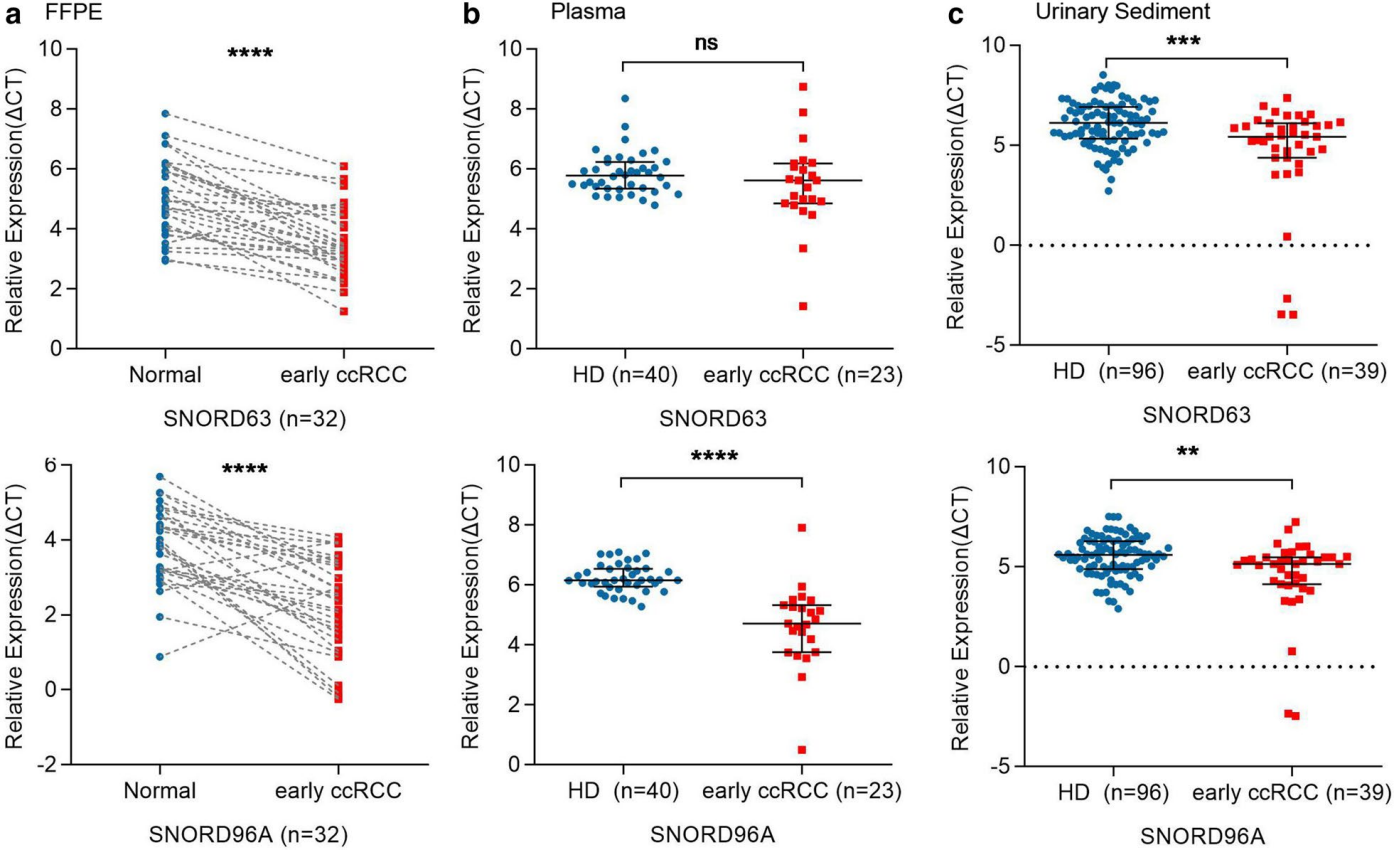
The expression of SNORD63 and SNORD96A in formalin fixed paraffin embedding (FFPE), plasma and urinary sediment (US) for early stage ccRCC patients. **a** The differentially expression of SNORD63 and SNORD96A in FFPE in 32 early stage ccRCC patients compared with adjacent tissues (****P < 0.0001). **b** The differentially expression of SNORD63 and SNORD96A in plasma in 23 early stage ccRCC patients vs. 40 healthy donors (HD) (ns: no significance, ****P < 0.0001). **c** The differentially expression of SNORD63 and SNORD96A in US in 39 early ccRCC patients vs. 96 healthy donors (HD) (***P < 0.001, **P < 0.01)

In order to assess the early ccRCC diagnostic efficacy of SNORD63 and SNORD96A, their expressions were detected in FFPE, plasma and US between early stage ccRCC patients and healthy donors, respectively. As shown in Fig. 5a–c, they were upregulated significantly in FFPE and US of early-stage ccRCC patients compared to those of healthy donors. Besides, SNORD96A but not SNORD63 was elevated in plasma of early stage ccRCC patients.

**Fig. 6:**
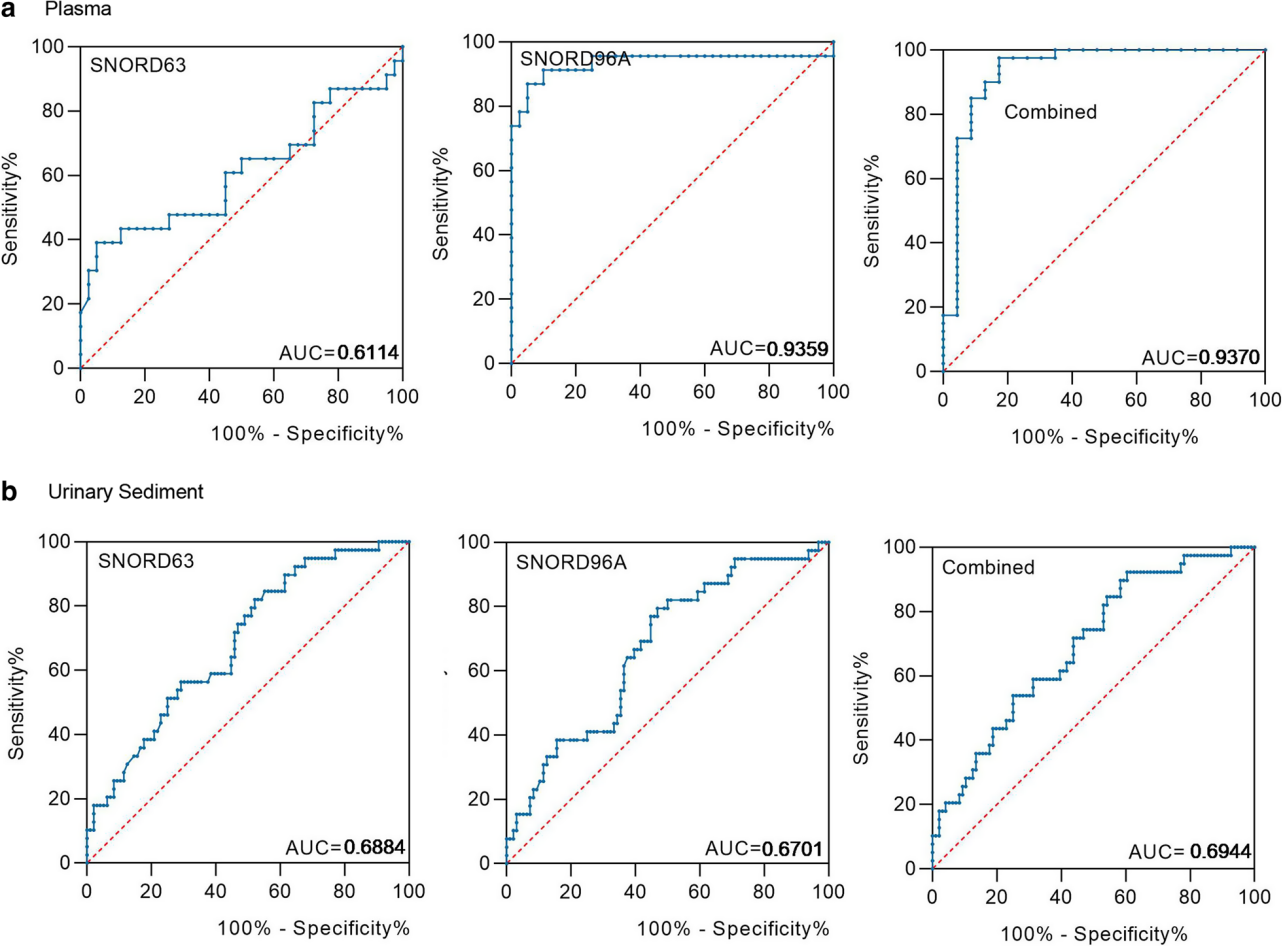
SNORD63 and SNORD96A as the early diagnostic biomarkers for ccRCC patients. **a** The areas under the curves (AUCs) of SNORD63 and SNORD96A in plasma for early ccRCC  were 0.6144 and 0.9359, respectively. The diagnostic performance for their combination demonstrated the AUC of 0.9370. **b** The areas under the curves (AUCs) of SNORD63 and SNORD96A in urinary sediment (US) were 0.6884 and 0.6701, respectively. The diagnostic performance for their combination demonstrated the AUC of 0.6944

Next, we calculated the early diagnostic efficacy of the two snoRNAs for ccRCC. In plasma, the AUCs of SNORD63 and SNORD96A were 0.6144 with 95% sensitivity and 39.1% specificity, 0.9359 with 95% sensitivity and 87% specificity, respectively. Their combination possessed AUC of 0.9370 with a relative sensitivity of 82.6% and a relative specificity of 97.5% (Fig. 6a); In US, the AUCs of SNORD63 and SNORD96A were 0.6884 with 47.9% sensitivity and 82.1% specificity, 0.6701 with 53.1% sensitivity and 79.5% specificity, respectively (Fig. 6b, right and mid). Their combination possessed AUC of 0.6944 with a relative sensitivity of 92.3% and a relative specificity of 39.6%, as well (Fig. 6b, left).

## Discussion

Previous studies demonstrated that SNORA70B, SNORD93, SNORD12B, SNORA59B, SNORA2, SNORD116-2 were abnormal expression in ccRCC [9], indicating the important role of snoRNAs in the development of ccRCC and the potential value of snoRNAs as diagnostic biomarkers for ccRCC patients. In the current study, we screened out SNORD63 and SNORD96A, then validated their increased expression in ccRCC.

Recently, accumulating evidence have demonstrated disturbances in the expression of snoRNAs in various cancer, indicating snoRNAs contribute to tumorigenesis and tumor development. In current research, we demonstrated SNORD63 and SNORD96A were increased in ccRCC significantly and stably, as evidence from the study on TCGA database, FFPE, US and plasma samples, although their functional roles had not been clarified in ccRCC. SNORD63 is located in chromosome 5q31.2 with a length of 68 nt and resides in intronic of HSPA9B, and functioned complementally by directing 2′-O-methylation of A531 in 28S rRNA residues [13]; Meanwhile, SNORD96A, located in chromosome 5q35.3 with a length of 72 nt and resides in intronic of GNB2L1, has been predicted to direct Gm75 (5.8S rRNA) [14], involved in cartilage ageing and osteoarthritis [15]. We believe that SNORD63 and SNORD96A play crucial role in the tumorigenesis and development of ccRCC, but the underlying molecular mechanisms needs further studies to illuminate.

As described above, snoRNAs are stably expressed and measurable in body fluids, empowering them the potential as biomarkers in various cancer types, such as NSCLC [16–20], CRC [21–24], breast cancer [25–27] and prostate cancer [28–30]. In current study, we demonstrated that SNORD63 and SNORD96A acted as the non-invasive diagnostic biomarkers for ccRCC even for early stage ccRCC. Notably, the diagnostic performance of SNORD63 and SNORD96A exerted quite differently, SNORD63 possessed higher diagnostic efficiency in US than in plasma; whereas SNORD96A acted on the contrary. It was quite difficult to address this issue. Because it might neither attribute to their stability and position in plasma since no differences were observed between these two snoRNAs (Fig. 2), nor to their subcellular localization in cancer cell since they distributed both in nucleus and cytoplasm in ccRCC cells (data no shown). Our data pointed the different biologic feathers of the two snoRNAs in ccRCC but needed further exploration.

However, several limitations should be carefully considered in the present study. First, the total sample sizes in current study were small and might lack statistically vigorous power. Long-term clinical follow-up data were also absent, which currently limit the ability to explore the prognostic values of the two snoRNAs; In addition, due to the limitation of snoRNA database, only TCGA database which was used to test snoRNA expression is main limitation of the study. Moreover, paired US and plasma from the same donor was difficult to collect, thus we failed to analyze the diagnostic performance of the two snoRNAs in US combined with in plasma.

## Conclusion

Collectively, we screened out SNORD63 and SNORD96A, then validated they were increased in ccRCC significantly and stably, as evidence from the study on TCGA, SNORic database, FFPE, renal cancer cell lines, plasma and US, implying the potential role in tumorigenesis. Importantly, SNORD63 in US and SNORD96A in plasma possessed the favorable diagnostic efficiency, suggesting that aberrant expression of SNORD63 and SNORD96A act as a diagnostic promising biomarker for ccRCC.

## Supplementary Information

**Additional file 1: Table S1. **Primers sequence involved.

**Additional file 2:** **Figure S1.** The expression of SNORD104, SNORD111, SNORD10 and SNORD95 in formalin fixed paraffin embedding (FFPE) and urinary sediment (US) for 24 ccRCC patients and healthy donors (HD). A. The differentially expression of SNORD104, SNORD111, SNORD10 and SNORD95 in FFPE in 24 ccRCC patients compared with adjacent tissues (***P < 0.001, **P < 0.01). B. The differentially expression of SNORD104, SNORD111, SNORD10 and SNORD95 in US in 24 ccRCC patients vs. 24 healthy donors (HD) (ns: no significance).

**Additional file 3:** **Figure S2.** Expression of SNORD63 and SNORD96A in renal cell cancer cells. A. Expression of SNORD63 in ACHN, 786-O, OS-RC-2 and Caki-1 cells. B. Expression of SNORD96A in ACHN, 786-O, OS-RC-2 and Caki-1 cells

## Data Availability

The datasets used and analyzed during the current study are available from the corresponding author on reasonable request.

## Abbreviations

ccRCC: Clear cell renal cell carcinoma
RCC: Renal cell carcinoma
SnoRNAs: Small nucleolar RNAs
nt: Nucleotides
US: Urinary sediment
NSCLC: Non-small cell lung cancer
NGS: Next-generation sequencing
TCGA: The Cancer Genome Atlas
FFPE: Formalin fixed paraffin embedding
AJCC: American Joint Committee on Cancer
WHO: World Health Origanization
cDNA: Complementary DNA
SPSS: Statistical Product and Service Solutions
ROC: Receiver operator characteristic
AUC: Area under curve
ns: No significance
HD: Healthy donors
MV: Microvesicles
