# Supplementary material for: SNORD63 and SNORD96A as the non-invasive diagnostic biomarkers for clear cell renal cell carcinoma
Source: Cancer Cell Int. 2021 Jan 18;21:56. doi: 10.1186/s12935-020-01744-4 (PMC7812721; doi:10.1186/s12935-020-01744-4)
Supplement: Supplementary file 1 — Additional file 1: Table S1. Primers sequence involved. [file 12935_2020_1744_MOESM1_ESM.docx]

**Table S1: Primers sequence involved.**

| **Gene** | **Forward Primer** | **Reverse Primer** |
| --- | --- | --- |
| ***SNORD104*** | GGCCTGCTGTGATGACATTCC | GGCTCAGACTCCAGTTCGCATC |
| ***SNORD111*** | CAGCCTGAAATGATGACTCTTTAA | AGGCAAAAACTGTGTCCAGAGA |
| ***SNORD10*** | CATGCGTGTCATCTGAGCCTCTG | TCCCGCCAGGCAGACAACAG |
| ***SNORD95*** | CGGTGATGACCCCAACATGCC | GCTCAGAAACAGCCTCTGGATTTC |
