# Supplementary figures and images for: SNORD63 and SNORD96A as the non-invasive diagnostic biomarkers for clear cell renal cell carcinoma
Source: Cancer Cell Int. 2021 Jan 18;21:56. doi: 10.1186/s12935-020-01744-4 (PMC7812721; doi:10.1186/s12935-020-01744-4)

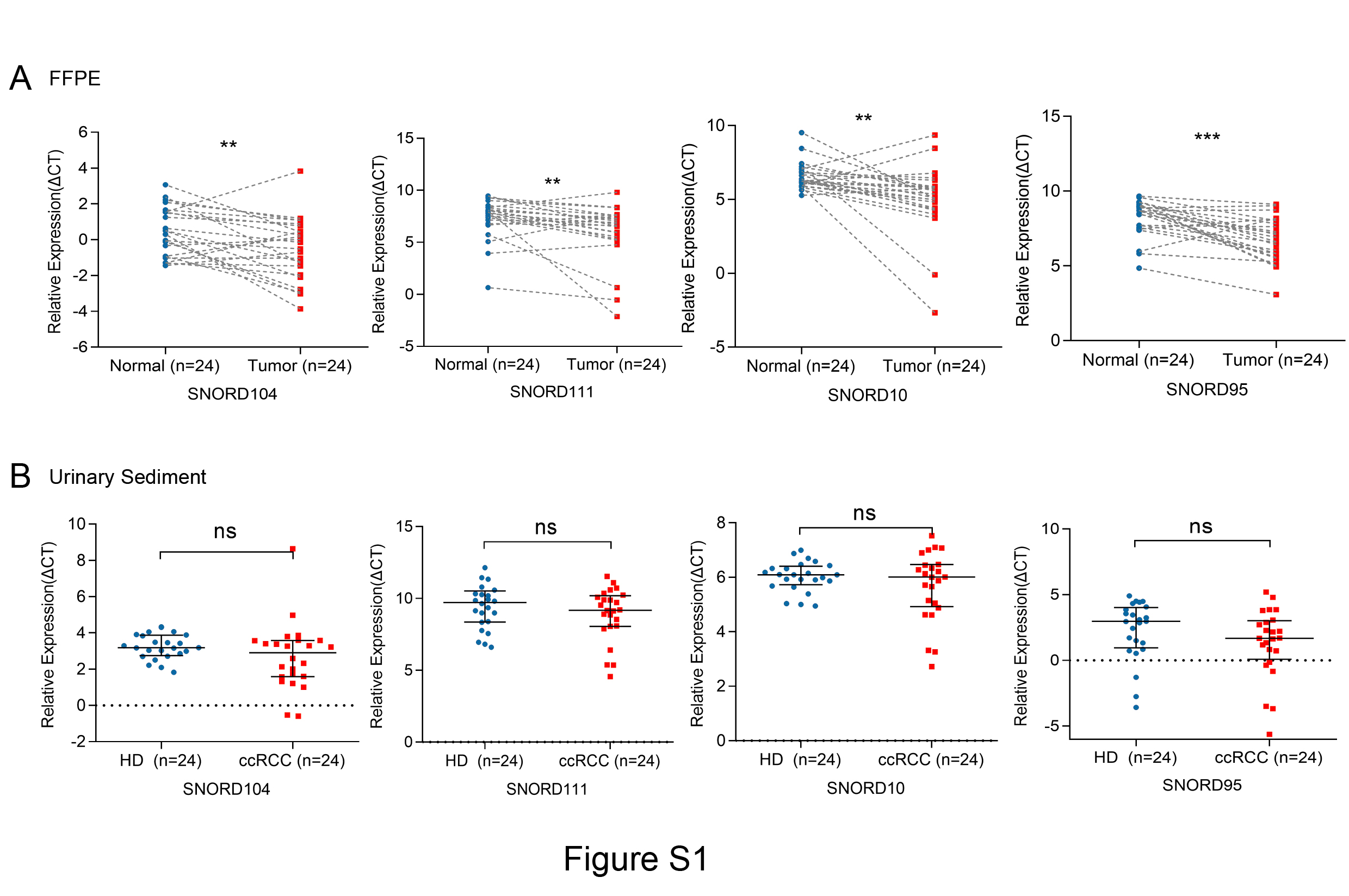

Supplement: Supplementary file 2 — Additional file 2: Figure S1. The expression of SNORD104, SNORD111, SNORD10 and SNORD95 in formalin fixed paraffin embedding (FFPE) and urinary sediment (US) for 24 ccRCC patients and healthy donors (HD). A. The differentially expression of SNORD104, SNORD111, SNORD10 and SNORD95 in FFPE in 24 ccRCC patients compared with adjacent tissues (***P < 0.001, **P < 0.01). B. The differentially expression of SNORD104, SNORD111, SNORD10 and SNORD95 in US in 24 ccRCC patients vs. 24 healthy donors (HD) (ns: no significance). [file 12935_2020_1744_MOESM2_ESM.jpg]

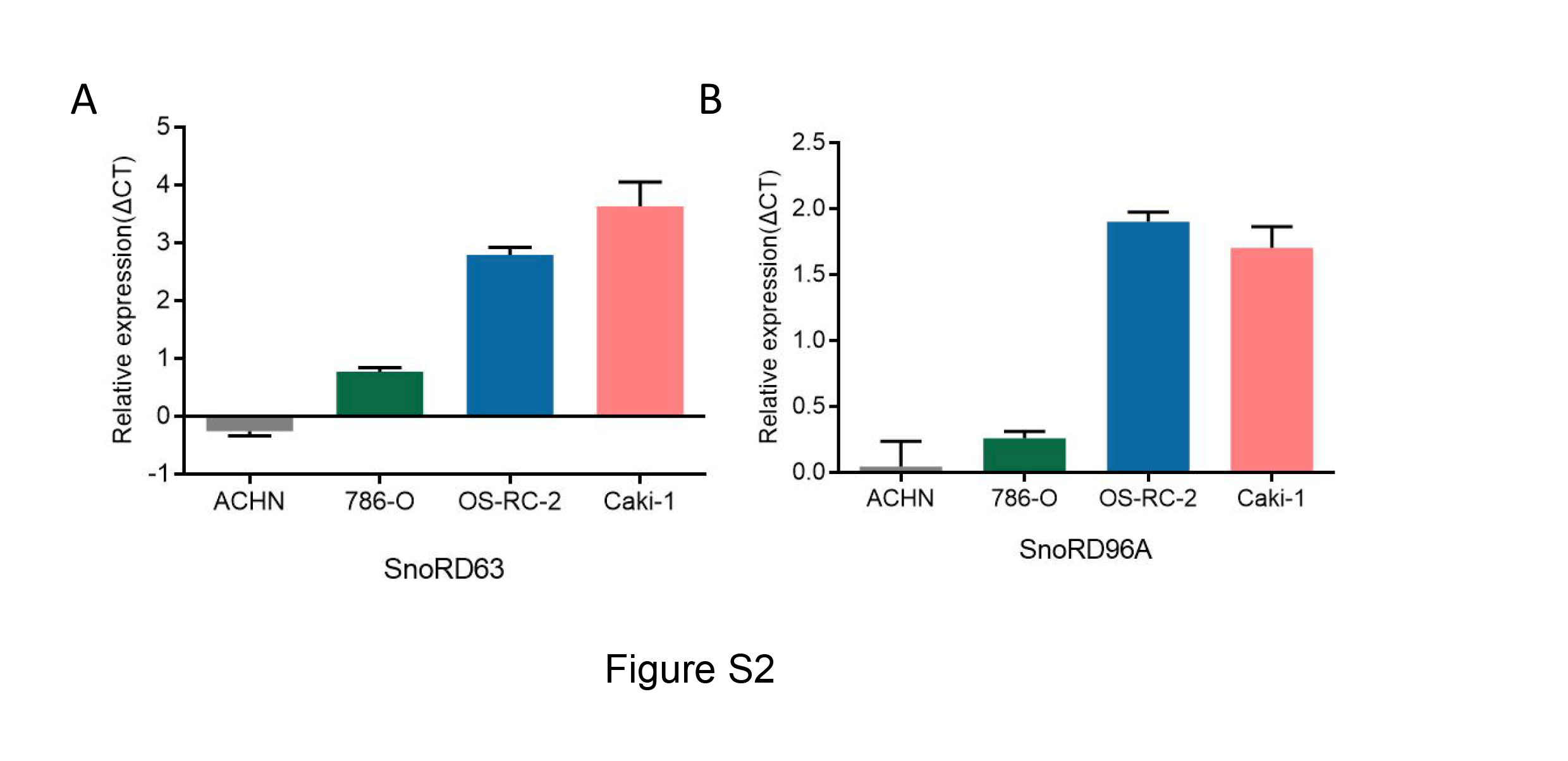

Supplement: Supplementary file 3 — Additional file 3: Figure S2. Expression of SNORD63 and SNORD96A in renal cell cancer cells. A. Expression of SNORD63 in ACHN, 786-O, OS-RC-2 and Caki-1 cells. B. Expression of SNORD96A in ACHN, 786-O, OS-RC-2 and Caki-1 cells [file 12935_2020_1744_MOESM3_ESM.jpg]
